# First record of the leafhopper genus Varicopsella Hamilton, 1980 (Hemiptera: Cicadellidae: Macropsinae) in China, with descriptions of a new subgenus and new species, a checklist, and a key to species

**DOI:** 10.1093/jis/14.1.125

**Published:** 2014-09-01

**Authors:** Hu Li, Ren-Huai Dai, Zi-Zhong Li

**Affiliations:** 1 Institute of Entomology, Guizhou University, Guiyang, Guizhou, and the Provincial Key Laboratory for Agricultural Pest Management of Mountainous Region, Guiyang, Guizhou 550025 P.R. China; 2 School of Biological Sciences & Engineering, Shaanxi University of Technology, Hanzhong, Shaanxi, and Bio-resources Key Laboratory of Shaanxi Province, Shaanxi University of Technology, Hanzhong, Shaanxi 723000 P.R. China

**Keywords:** Auchenorrhyncha, morphology, distribution

## Abstract

A new monobasic leafhopper subgenus, *Varicopsella* ( *Multispinulosa* ) Li, Dai, and Li, subgen. nov., of the subfamily Macropsinae (Hemiptera: Auchenorrhyncha: Membracoidea: Cicadellidae) is proposed to accommodate *Varicopsella* ( *Multispinulosa* ) *hamiltoni* Li, Dai, and Li, sp. nov.from Guangxi province of China. The new subgenus and new species are described and illustrated. They can be distinguished mainly by characteristics of the fore wings with two anteapical cells; weak dorsoventrally flattened body; aedeagal shaft with paired apical processes on ventral margin; and the shape of the dorsal connective. An updated checklist and an illustrated key for identification of the species of *Varicopsella* along with geographical distributions of the species are given. 
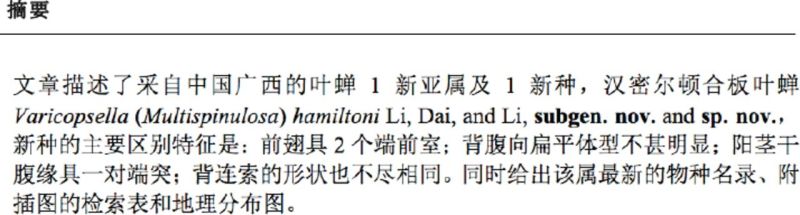

## Introduction

The oriental leafhopper genus *Varicopsella*Hamilton, 1980 (Hemiptera: Auchenorrhyn-cha: Membracoidea: Cicadellidae: Macropsinae) was established by Hamilton (1980) for six species from the Oriental region (Philippines and Borneo), with *Macropsis breakeyi*Merino, 1936 as its type species by original designation. One year later, Viraktamath (1981), described a new species from India. Up to now, no other species was described in this genus. Considering the vastland and abundant resources, macropsine fauna are likely to be richer in southern China,including the genus *Varicopsella* .

Deposited in our collection from Guangxi Province of China (included in the Oriental Region), two specimens were found that bear the distinct generic characteristics of *Varicopsella* : the fused sclerites between lora and frons on face, and the independently evolved dorsal connectives and pygofer processes in males. These specimens have been identified as a new species of *Varicopsella* , and on the basis of the distinctive feature that the fore wings of the new species have two anteapical cells, we propose to place it in a new subgenus. The identification was verified by K. G. A. Hamilton, pers. comm. (Research Branch, Agriculture and Agri-Food Canada).

In this paper, the genus *Varicopsella* is reported for the first time from China based on the new species. The new taxa ( *Varicopsella* ( *Multispinulosa* ) *hamiltoni* Li, Dai, and Li, subgen. nov.and sp. nov.from Guangxi province (China) are described and illustrated. Photographs of imago and illustrations of the male genitalia are provided, and an illustrated key to the species of the genus based on the original descriptions and illustrations, with a map of distribution of the genus based on the primary, data are given.

## Materials and Methods

Morphological terminology used in this work follows Hamilton (1980) and of the rows of setae on the legs follows Rakitov (1998) . The type specimens examined here are deposited in the Institute of Entomology, Guizhou University, Guiyang, China (GUGC).

### Nomenclature

This paper and the nomenclature it contains have been registered in ZooBank. The LSID number is:

urn:lsid:zoobank.org :pub:F8C13A75-E433-4DA8-8534-1144943E3686

### Systematics

Subfamily Macropsinae Evans, 1938 Genus *Varicopsella*Hamilton, 1980

Subgenus *Varicopsella* ( *Varicopsella* )Hamilton, 1980

*Varicopsella* Hamilton, 1980: 900. Type species: *Macropsis breakeyi* Merino, 1936.

### Remarks.

The genus *Varicopsella* can be distinguished from other Macropsinae mostly by the following characteristics: fused sclerites between lora and frons on face, and the unique male dorsal connectives each formed by two articulating parts with ventral pair appressed or fused with each other and dorsal ones produced as variously-shaped projections among species. The unique distinctive feature of the nominate subgeneric *Varicopsella* ( *Varicopsella* ) is fore wings with three anteapical cells.

Distribution.Oriental, distributed in Philippines, Borneo, and India ( Hamilton 1980 ).

Subgenus *Varicopsella (Multispinulosa)* Li, Dai, and Li, subgen. nov.

Type species: *Varicopsella (Multispinulosa) hamiltoni* Li, Dai and Li, sp. nov.

**Figure 1-6.**
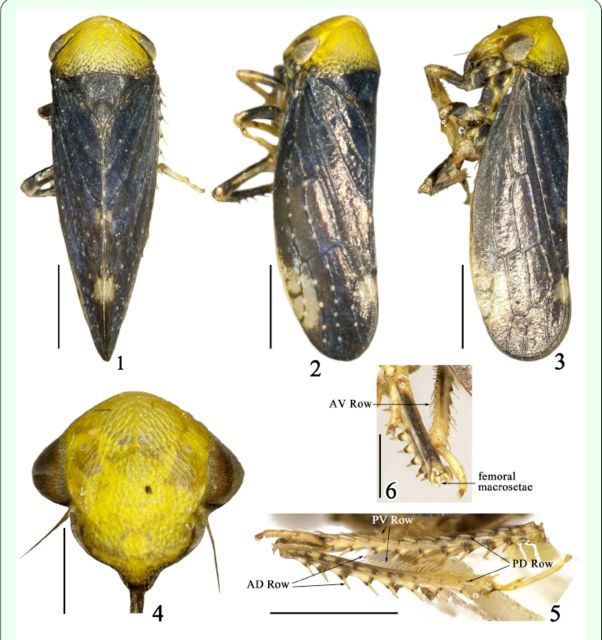
*Varicopsella (Multispinulosa) hamiltoni* sp. nov.(male). (1) Habitus, dorsal view; (2) Dorsolateral view; (3) Lateral view; (4) Face; (5) Hind tibia of both leg, caudal view; (6) Hind femur of left leg, lateral view. Scale bar = (1-3, 5) 1.0 mm, (4, 6) 0.5 mm.

Description.Body ( Fig. 1 -4) typical wedge-shaped; head, face, pronotum, and scutellum strongly striated.

Head ( Fig. 1 ), in dorsal view, clearly arcuate anteriorly, and width across eyes equal to pronotum, crown shorter medially than besides eyes. Face ( Fig. 4 ), across eyes slightly wider than long, obviously co-planar, with lora fused to frons, lacking clear sutures between them, dorsal part with median carina, along it bilaterally with slant striations ended below ocelli; distance between ocelli about 5xlonger than that of ocellus to adjacent eye. Pronotum ( Fig. 1 ) 2.1x as long as wide, anterior margin strongly prominent, posterior margin slightly concave medially, almost straight, surface with strongly oblique striations along median carina. Scutellum ( Fig. 1 ) nearly triangular, with 1.2x as long as pronotum, strongly striated except bilateral corners with relatively smooth surface, coalescent suture between mesonotum and secutellum distinct, arcuate. Forewings opaque ( Fig. 1 -3), with 2 anteapical cells, veins protruding. Hind femoral macrosetae ( Fig. 6 ) 2+1; hind tibia ( Fig. 5 ) with 8 macrosetae on AD row, 5 on AV row, 11 on PD row, dense and slender on PV row.

**Figure 7-15.**
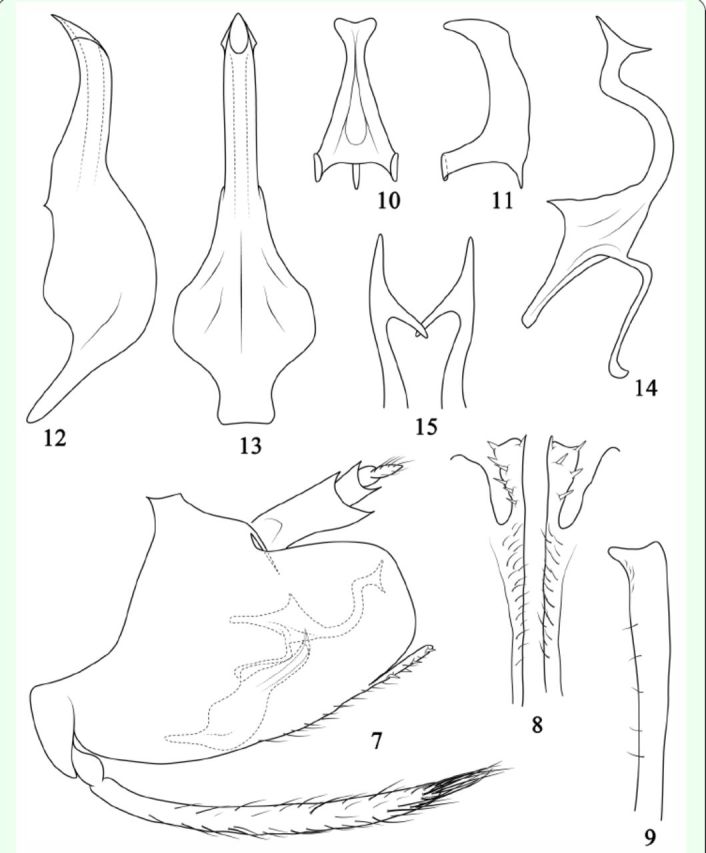
Male genitalia of *Varicopsella (Multispinulosa) hamiltoni* sp. nov.(7) Pygofer lobe and subgenital plate, lateral view; (8) Pygofer processes and spines on ventral margin; (9) Apex of style, lateral view; (10) Connective, dorsal view; (11) Same, lateral view; (12) Aedeagus, lateral view; (13) Same, ventral view; (14) Dorsal connective, lateral view; (15) Dorsal end of dorsal connective, caudal view.

Male genitalia.Pygofer ( Fig. 7 -8) broad basally, ventral margins with some setae subapically and produced into triangular processes with small spines on surface. Subgenital plates ( Fig. 7 ) slender with scattered setae. Styles ( Fig. 9 ) slender with marginal setae, nearly angled on basal 0.45, slightly intumescent subapically, apex nearly rectangular on ventral margin. Connective ( Fig. 10 -11) similar to others of genus. Aedeagus ( Fig. 12 -13) simple, broad basally, shaft sinuated, shaft apex sharp, dorsally directed, with pair of processes on ventral margin, dorsal apodeme weakly developed, ventral margin concave in middle; gonopore apical on ventral margin. Dorsal connectives ( Fig. 14 -15) strongly developed, ventral portions appressed with each other, dorsal portions slender, sinuate, with dorsal end bilobed.

Distribution.Oriental, China (new record of genus), Guangxi Province.

Remarks.The new subgenus can be easily distinguished from the other *Varicopsella* subgenus by the fore wings with two anteapical cells; weakly flattened external form of the body; unique male aedeagal shaft with paired apical processes on ventral margin; and shape of the dorsal connective.

Etymology.The subgenus name is derived from the Latin words *“multi-”* and *“spinu-losus”* because of the several spines on the ventral margin projections of the pygofer lobe. The gender of the subgenus is feminine.

*Varicopsella (Multispinulosa) hamiltoni* Li, Dai, and Li sp. nov. ( Fig. 1 -15, 21)

Measurements.Body length including teg-mina, *$,* 3.9-4.1 mm.

Body color.Background color ( Fig. 1 -4) yellowish-green, striations on head, face, and pronotum with same tint as background except posterior margin of pronotum with brown markings. Head, face, and pronotum ( Fig. 1 -4) yellowish-green. Eyes brown, with some what slightly and occasionally red crown; ocelli brightly lucid; antennal fossa, scape, pedicel, and flagellum successively vary from yellowish green to brown; terminal anteclypeus, beak, and outer margins of both lora yellowish-brown to brown. Scutellum ( Fig. 1 ) fully black except bilateral margins slightly yellowish-brown medially. Fore wings ( Fig. 1 -3) dark brown, except terminal regions of veins 1A, distal claval suture and outer anteapical part with brown maculae successively enlarged in size, veins clearly spotted with white. Legs ( Fig. 5 -6) brown with black maculae.

Structural morphology.As in generic description.

Male genitalia.Pygofer ( Fig. 7 -8), in lateral view, broad basally, truncate caudally, in ventral view, ventral margins with some setae subapically and elongate to nearly triangular processes slightly exceeding pygofer length, and with several small spines on surface. Subgenital plate ( Fig. 7 ), in lateral view, rodlike, scattered with hair-like setae, apex with bunched long setae, slightly projecting beyond pygofer. Style ( Fig. 9 ), slender, margined with some setae, nearly angled on basal 0.45, slightly inflated subapically, apex angular with dorsally twisted digitation. Connective ( Fig. 10 -11), in dorsal aspect, clearly longer than wide, with anterior margin strongly wider than posterior margin, anterior margin excavated medially, and with finger-like process in middle, both lateral arms slender and blend dorsally. Aedeagus ( Fig. 12 -13) tubular, broad basally, shaft apex sharpened, with pair of triangular processes on ventral margin; in lateral aspect, dorsal apodeme weakly developed, shaft sinuated, ventral margin concave medially, tip twisted to dorsum; in ventral aspect, shaft nearly parallel margined; gonopore on ventral margin, apical. Dorsal connective ( Fig. 14 -15) strongly developed, ventral portions appressed with each other, with dorsal end bearing two digitations; in lateral view, dorsal portions long, sinuate; in caudal view, ventral paired digitations ventrally directed to each other.

**Figure 16-20.**
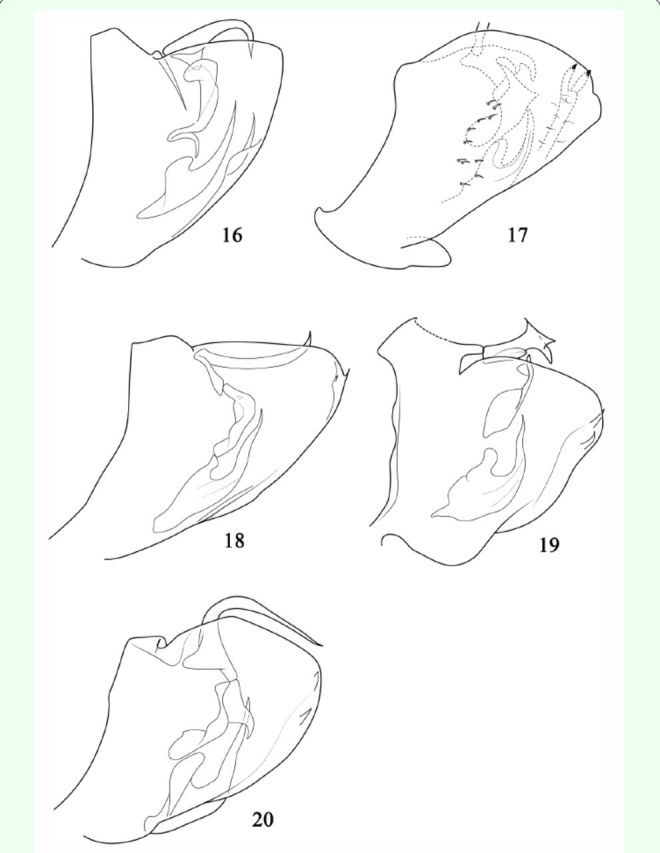
Male genitalia of *Varicopsella* species: pygofer lobe, aedeagus and dorsal connective, lateral view. (16) *Varicopsella* ( *Varicopsella* ) *breakeyi* ; (17) *V* . ( *V* .) *elegans* ; (18) *V* . ( *V* .) *luzonensis* ; (19) *V* . ( *V* .) *obtusa* ; (20) *V* . ( *V* .) *otanesi* . (16, 18–20. After Hamilton, 1980; 17. After Viraktamath, 1981 ).

Female.Unknown.

Materials examined.Holotype, *S,* CHINA: Guangxi Province, Longsheng County, Huaping National Natural Reserve, 18. V. 2012, collected by Li Hu; Paratype, 1 *$,* CHINA: Guangxi Province, Baise City, Tian-lin County, Langping Village, 23. IV. 2012, collected by Yang Weicheng.

**Figure 21.**
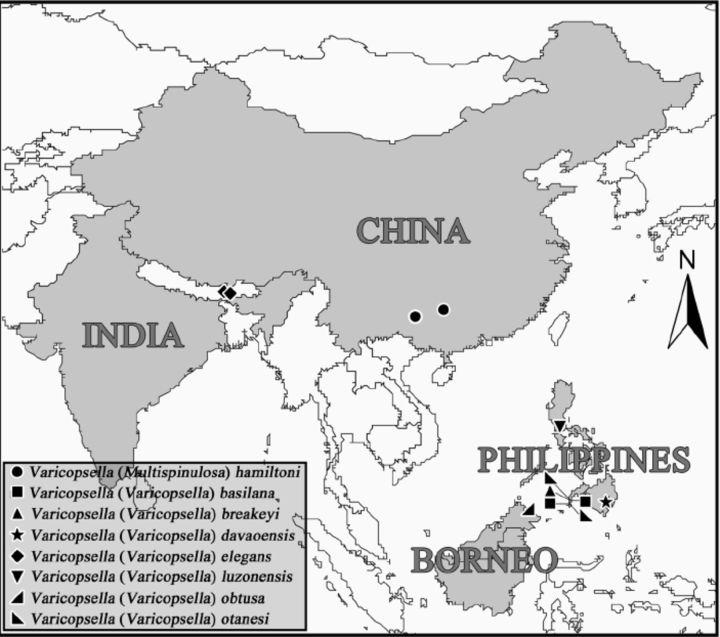
Map showing distribution of species of *Varicopsella.*

Distribution.Guangxi prov. (Huaping, Langping), China ( Fig. 21 ).

Etymology.This species is named in honor of K. G. A. Hamilton for excellent contributions to the Auchenorrhyncha systematics and invaluable help to the first author.

### Updated checklist and distributions of the species of genus *Varicopsella*

*Varicopsella (Multispinulosa) hamiltoni* sp. nov.

Distribution. China (Huaping, Langping).

*Varicopsella (Varicopsella) basilana* (Merino) *Macropsis basilana*Merino, 1936 : 323. *Varicopsella basilana,* Hamilton, 1980: 900. Distribution. Philippines (Basilan, Mindanao).

*Varicopsella (Varicopsella) breakeyi* (Merino) *Macropsis breakeyi*Merino, 1936 : 320. *Varicopsella breakeyi,*Hamilton, 1980 : 900. Distribution. Philippines (Mindanao).

*Varicopsella (Varicopsella) davaoensis* (Merino)

*Macropsis davaoensis* Merino, 1936 : 325. *Varicopsella davaoensis* , Hamilton, 1980 : 900.

Distribution. Philippines (Mindanao).

*Varicopsella* ( *Varicopsella* ) *elegans* Viraktamath

*Varicopsella elegans* Viraktamath, 1981 : 306. Distribution. India (Himalayas).

*Varicopsella* ( *Varicopsella* ) *luzonensis* (Merino)

*Macropsis luzonensis* Merino, 1936 : 324. *Varicopsella luzonensis* , Hamilton, 1980 : 900. Distribution. Philippines (Luzon).

*Varicopsella* ( *Varicopsella* ) *obtusa* Hamilton *Varicopsella obtusa*Hamilton, 1980 : 919. Distribution. Borneo (Sandakan).

*Varicopsella* ( *Varicopsella* ) *otanesi* (Merino) *Macropsis otanesi*Merino, 1936 : 323. *Varicopsella otanesi* , Hamilton, 1980 : 900. Distribution. Philippines (Basilan, Mindanao).

### Key to the Subgenera and Species of Male *Varicopsella*

The *Varicopsella* species are keyed based on the variations of male genitalia, therefore two species only known by the female, *V* . ( *V* .) *basilana* ( Merino, 1936 ) and *V* . ( *V* .) *davaoensis* ( Merino, 1936 ), are excluded from the key.

1. Body form not strongly flattened dorsoventrally ( Fig. 3 ); fore wings with 2 anteapical cells ( Fig. 3 ); aedeagal shaft with pair of triangular processes apically ( Fig. 13 )… *V* . ( *Multispinulosa* ) *hamiltoni* subgen. nov. and sp. nov.

– Body form strongly flattened dorsoventrally; fore wings with 3 anteapical cells; aedeagal shaft without pair of triangular processes apically ( Fig. 16 –20)………... *V* . ( *Varicopsella* ) 2

2. Pygofer with one small, inturned spine on each lobe ( Fig. 16 ); ventral part of dorsal connective fused to each other ( Fig. 16 )……… *V* . ( *Varicopsella* ) *breakeyi*

– Pygofer with two small apical spines or teeth on each lobe ( Fig. 17 –20); ventral part of dorsal connective appressed with each other ( Fig. 17 –20)…………………………………3

Dorsal connective short with tip bifid or trifid ( Fig. 17 , 19)…………………………...4 – Dorsal connective slender with tip sharp, not furcated ( Fig. 18 , 20)………………………..5

Dorsal connective with tip trifid ( Fig. 19 ); aedeagal shaft sinuate ( Fig. 19 )……………… ……………………... *V* . ( *Varicopsella* ) *obtuse* – Dorsal connective with tip bifid ( Fig. 17 ); aedeagal shaft arched but not sinuate ( Fig. 17 )………………… *V* . ( *Varicopsella* ) *elegans*

Pygofer with two small spines on each lobe ( Fig. 20 ); dorsal connective elongate process with dorsal end directed ventrally ( Fig. 20 )…………………. *V* . ( *Varicopsella* ) *otanesi* – Pygofer with two extremely tiny spines on lobe ( Fig. 18 ); dorsal connective elongate process with dorsal end directed dorsally ( Fig. 18 )……………… *V* . ( *Varicopsella* ) *luzonensis*
